# Thoughts and musings from the new International Population Data Linkage Network (IPDLN) co-directors: Building on our strengths in the next two years of the IPDLN (2017-2019)

**DOI:** 10.23889/ijpds.v1i1.403

**Published:** 2017-04-19

**Authors:** Michael Schull, William Ghali

**Affiliations:** 1 Institute for Clinical Evaluative Sciences (ICES); 2 University of Calgary

The beginning of 2017 triggered a change in leadership at the International Population Data Linkage Network (IPDLN), as Professor David Ford's two year term as director of the network wound down, and the baton was handed on to new Canadian co-leadership, from the Institute for Clinical Evaluative Sciences in Ontario, and the O'Brien Institute for Public Health at the University of Calgary. David Ford's success and energy during his term were such that our two organizations decided we needed to team up to jointly direct the IPDLN for the next two years. This was endorsed by the IPDLN membership in the lead up to the August 2016 IPDLN conference in Wales, and we are delighted to co-direct this growing network.

First off, we want to thank David for his outstanding leadership of the IPDLN over the last two years. He and his team put on an excellent conference in Swansea, with record breaking attendance, excellent presentations, thought provoking plenaries and wonderful facilities and social events that made the conference a huge success. Even the weather cooperated, as the sun shone most of the time (well done David!). In addition, membership of the IPDLN has grown substantially in the last two years, both in number of members and number of different countries represented. David played a key role in the launch of a new journal for our network, the International Journal of Population Data Science (IJPDS), with an editorial board comprised of several IPDLN members, and with Associate Professor Kerina Jones (Swansea University) as founding editor-in-chief.

The IPDLN remains a young organization, having had its inaugural conference only in 2008, and we have come a long way in that time. Each subsequent biennial conference has grown in size, sophistication and quality of content, and our membership has steadily increased. In addition, new collaborations between IPDLN researchers and member organizations are continuing to develop. External trends, such as widespread interest in the possibilities of "Big Data" make the work and expertise of IPDLN members more relevant than ever. Common challenges across our countries, such as the perpetual concerns around quality and sustainability of our health and social care systems, make the IPDLN's ability to foster cross-jurisdiction research and evidence more important than ever.

We are committed to building on David's work and that of the directors before him to further the aims of the IPDLN. As a first step, we have established an interim executive committee, consisting of the current IPDLN co-directors and leaders of the organizations that have previously led the network (James Semmens, Curtin University, Kim McGrail, University of British Columbia, David Ford, Swansea University). Its purpose is to provide strategic advice on the overall direction and deliverables of the IPDLN. The interim executive committee will serve until the next conference in 2018, at which time a new director and a new executive committee will be elected by the membership.

One key deliverable will be another outstanding IPDLN conference, and we are pleased to announce it will take place in Banff, Alberta, from September 12-14, 2018. Please mark your calendars now and plan to attend. Dr Hude Quan and Dr Astrid Guttmann have agreed to lead the Scientific Programme Committee, and we will be providing more information on opportunities to participate in the work of that committee in due course. In addition, we aim to continue to increase IPDLN membership in terms of number and geography, especially among countries that are not (or only modestly) represented. We will also help IPDLN member institutions to create more opportunities for collaboration, cross-jurisdictional studies, and shared tools and learning.

The scientific focus of our young network is evolving, reflected in the recent decision to substitute "population data" for "health data" in the network's name. This was welcome in that it reflected the fact that many of our members study more than health data or health outcomes, but it might make it harder to describe the mission of the network. Similarly, how the IPDLN can better contribute to shaping a conversation around our collective mission, advance science between conferences, and ensure that the network and its members are collectively seen as leaders internationally, are questions being explored and will inform the network development plan.

The IPDLN is well-positioned to shape the global scientific agenda in emerging areas such as `population data science' and/or `Big Data'. Such terms, describing domains of scientific inquiry, mean different things to different people. Our network and its broad and interdisciplinary international membership can help to define what these areas are, by identifying high priority population data science questions and opportunities for ambitious collaborative efforts on a global scale.

These are exciting times for the IPDLN and its members, as in this digital health information age the potential for our network is limitless. In our early days as co-directors, we welcome your input in response to these ideas on the role of the IPDLN (please email us with your ideas and thoughts). We are honoured to have the opportunity to co-lead the IPDLN for the next two years, and recognize that our efforts will be made easier because we are building on the existing strengths of the network and its members.

## Please email us your ideas and thoughts on the role of IPDLN to:

Michael Schull at Michael.Schull@ices.on.ca

William Ghali at wghali@ucalgary.ca

